# Platelet to lymphocyte ratio in the healthy population of Vojvodina

**DOI:** 10.5937/jomb0-60091

**Published:** 2026-01-06

**Authors:** Tanja Šašić-Ostojić, Stanislava Nikolić, Maša Sladojević, Velibor Čabarkapa, Dušan Sedlarević, Dragana Žuvić

**Affiliations:** 1 University Clinical Centre of Vojvodina, Center of Laboratory Diagnostics, Novi Sad; 2 Faculty of Medicine, University of Novi Sad, Novi Sad

**Keywords:** complete blood count, laboratory testing, platelet to lymphocyte ratio, reference values, kompletna krvna slika, laboratorijsko testiranje, odnos trombocita i limfocita, referentne vrednosti

## Abstract

**Background:**

The platelet-to-lymphocyte ratio (PLR) is a simple laboratory index that can be applied in the diagnosis and follow-up of various diseases, particularly those with a primary or accompanying systemic inflammatory component. However, its clinical utility is limited by the absence of established reference intervals in the general population. This study aimed to determine the reference interval for PLR.

**Methods:**

This retrospective observational study included 4,672 adults who underwent regular systematic check-ups consisting of clinical examinations and basic laboratory analyses, including a complete blood count (CBC). The PLR reference interval was calculated using Clinical and Laboratory Standards Institute (CLSI) guidelines, which estimate percentiles and their 95% confidence intervals (CI). Verification of the reference interval was performed in a group of 95 healthy adults, matched by age and sex to the study cohort.

**Results:**

The mean age of participants was 43± 10 years, with a predominance of males (83.7% ). The median PLR was 109 (25th percentile: 90; 75th percentile: 131). The lower limit of the reference interval was 62 (95% CI: 61-63), and the upper limit was 194 (95% CI: 188-198). Female participants had significantly higher PLR values compared with males (P &lt; 0.0001), while elderly participants had significantly lower PLR values compared with the middle-aged and younger groups (P= 0.019). The determined reference interval was 70-231 for females and 61-183 for males. PLR values did not differ significantly between the primary study group and the validation group [108.0 (87-136) vs. 109.0 (90-131); P&gt; 0.05].

**Conclusions:**

In this representative sample, consisting primarily of young and middle-aged adults, the PLR reference interval was 70-231 for women and 61-183 for men. In individuals older than 65 years, PLR values may be lower.

## Abstract (sr)

**Uvod:**

Odnos trombocit-limfocit (PLR) predstavlja jednostavan laboratorijski indeks koji može imati značaj u dijagnostici i praćenju različitih oboljenja sa primarnom ili pratećom sistemskom inflamatornom komponentom. Međutim, zbog nedostatka referentnih intervala u opštoj populaciji, njegova klinička primena je ograničena. Cilj ove studije bio je da se utvrdi referentni interval za PLR indeks. Metodologija: Retrospektivna studija obuhvatila je podatke o 4.672 odrasla ispitanika koji su tokom sistematskog pregleda imali urađen klinički pregled i osnovne laboratorijske analize (kompletna krvna slika - KKS). Referentni interval za PLR određen je primenom Vodiča za kliničke i laboratorijske standarde, procenom percentila i njegovog 95% intervala poverenja (IP). Verifikacija je sprovedena u grupi od 95 klinički zdravih ispitanika oba pola, uparenih po polu i godinama sa ispitivanom grupom.

**Rezultati:**

Prosečna starost ispitanika bila je 43± 10 godina, sa dominacijom muškaraca (83,7% ). Medijana PLR je iznosila 109 (25. percentil: 90; 75. percentil: 131). Donja granica intervala bila je 62 (95% IP: 61-63), a gornja 194 (95% IP: 188-198). Žene su imale statistički značajno više vrednosti PLR-a u odnosu na muškarce (P &lt; 0,0001), dok su starije osobe imale značajno niže vrednosti u poređenju sa sredovečnim i mlađim grupama (P= 0,019). Utvrđeni referentni interval bio je 70-231 za žene i 61-183 za muškarce. Vrednosti PLR-a nisu se značajno razlikovale između inicijalne i verifikacione grupe [108 (87-136) vs. 109 (90-131), P&gt; 0,05].

**Zaključak:**

U reprezentativnom uzorku ispitanika, pretežno mlađih i sredovečnih osoba, referentni interval PLR-a iznosio je 70-231 za žene i 61-183 za muškarce. Kod osoba starijih od 65 godina vrednosti PLR-a mogu biti niže.

## Introduction

A complete blood count (CBC), a widely used basic haematology test, includes a hemogram and the differential count of white blood cells. Although CBC is most often used to diagnose anaemia, inflammatory processes, and immunodeficiency conditions, it has recently been determined that some parameters of CBC, such as the calculated neutrophil-to-lym- phocyte ratio (NLR) and platelet-to-lymphocyte ratio (PLR), are associated with activity, morbidity, and mortality in various diseases. PLR is a relatively new, simple and widely available index that can be useful in the detection of systemic inflammation of different clinical degrees (from subclinical to clinical) in a broad spectrum of diseases, such as acute cardiovascular events (heart failure, acute coronary syndrome) [1] [2], acute kidney injury, and malignancy [3]. PLR can be an indicator of inflammation and thrombosis equilibrium, as rapid proliferation of megakaryocytes, thrombocytosis, and platelet (PLT) aggregation tendencies are all present in inflammation. Besides, platelets are being differently activated depending on the stimulating signal and their functional role, and different levels of activation are necessary for the formation of a proper haemostatic clot or the recruitment of leukocytes. In the interaction of platelets and lymphocytes, alpha and delta granules are ed, causing T lymphocytes to be recruited to the site of inflammation or infection, a cruca crucialation of the initiationmmunthe Since affinity to inflammation and thrombosis are common denominator to all mentioned diseases, PLR index could be applied as reliable prediction parameter of inflammation response and mortality, as well as follow up parameter in these conditions [4].

Initially, PLR was introduced as a potential marker for determining inflammation in oncological disorders [5] [6]. The connection between inflammation and malignancy is bidirectional. An excessive or unregulated immune response may increase the risk of disease development, including oncogenesis. Approximately 20% of all carcinomas are associated with a chronic inflammatory response due to infection, exposure to irritants, or autoimmune diseases. Meanwhile, local inflammatory responses that occur in the microenvironment of the tumour itself could be potential targets for the prevention and treatment of malignancy [7]. Hence, the assessment of inflammatory response can have clinical relevance. To date, it has been established that the PLR index is a significant prognostic parameter in patients with non-microcellular lung carcinoma and a predictor of death caused by sepsis [8]. Many studies have shown that the PLR index is a long-term predictor of the main unfavourable cardiovascular events in patients with ST-segment elevation myocardial infarction (STEMI) [9]. In active rheumatoid arthritis and systemic lupus erythematosus [10] [11], PLR was found to be significantly higher compared to controls, and can be used to assess the disease activity. In the postoperative period, in patients with hepatocellular carcinoma after hepatectomy, an increased PLR index was connected to recidives [12]. In the study of Karadeniz et al., significantly higher values of PLR were observed in patients with stable and unstable chronic obstructive pulmonary disease [13].

In most published studies, different cut-off values of the PLR index have been defined for various diseases. The available literature provides no data on the unique reference value of PLR. Hence, the objective of this study was to determine the reference interval for the PLR index in the general population, as well as to identify the characteristics (gender and age) that are significant for the interpretation of index values.

## Materials and methods

In this retrospective observational study, the data of 4672 adult examinees, of both genders, were analysed. They all underwent systematic medical examination, performed by the Institute of Public Health of Vojvodina, between June 2016 and May 2017. The investigated data included demographic characteristics, comorbidities, anthropometric parameters, levels of systolic and diastolic arterial blood pressure and values of laboratory parameters - CBC and fasting glycemia. Consent to use medical documentation was obtained from the Ethical Committee of the Institute of Public Health of Vojvodina in Novi Sad. Examinees were of good general health with no known acute or chronic inflammatory, cardiovascular, kidney, liver, malignant or infectious diseases. Exclusion criteria included elevated levels of fasting glycemia, a known diagnosis of diabetes mellitus, and haematological diseases.

The control cohort group consisted of adults of both genders (age- and gender-matched, N=95) who presented to the University Clinical Centre of Vojvodina for laboratory examination. A subsequent review of medical data obtained from the clinical and laboratory information system confirmed the absence of any acute or chronic inflammatory processes, as well as cardiovascular, kidney, liver, malignant, or infectious diseases that could potentially influence the analysed ratio. All medical data were obtained with the consent of the Ethical Committee to verify the reference interval of PLR. The statistical analysis was conducted in accordance with the CLSI EP28-A3c guidelines for determining reference intervals [14]. The reference interval was defined as the range between the 25th and 75th percentiles in the observed population. Subsequent validation of the obtained reference interval was performed by testing an independent cohort for comparison with the established reference interval to assess its applicability and reliability in this population.

### Clinical measurement and analytical procedures

For all examinees, clinical measurement and laboratory analyses were performed in accordance with the following methodology:

Anthropometric parameters: Body height was measured using a Harpenden anthropometer (Holtain Ltd, Crovell, UK) with a precision of 0.1 cm. Body weight was measured in an upright position on an electronic scale with an accuracy of 0.1 kg. Body mass index (BMI) was calculated as the ratio of body mass and body height squared (kg/m^2^). Waist circumference was measured in the middle of the lowest rib and the tallest part of the femur and noted in centimetres (cm).

To all the examinees, arterial systolic and diastolic blood pressure (SBP and DBP) was measured by the Riva Rocci method.

Laboratory parameters: A complete blood count (CBC) was assessed using an automated haematology analyser, the Horiba ABX Micros 60, with Horiba ABX SAS (Montpellier, France) reagents, based on the flow cytometry principle. The coefficient of variation (CV) for white blood cells (WBC) was reported to be <2.5%, with linearity in the range of 0-100 × 10^3^/mm^3^. For platelets (PLT), the CV was <5%, and linearity ranged from 0-2200 × 10^3^/mm^3^.

In the second group of examinees, CBC was assessed using the automated haematology counter CELL-DYN Sapphire with commercially available reagents (Abbott Diagnostics, Illinois, USA). The CV for both WBC and PLT was reported to be <5%. Linearity was 0-250 × 10^3^/mm^3^ for WBC and 0-2000 × 10^3^/mm^3^ for PLT.

Using data obtained from the CBC (absolute platelet and lymphocyte counts), the PLT index was calculated by dividing the platelet count by the lymphocyte count.

Fasting blood glucose and C-reactive protein concentrations were measured using the biochemical analyser Architect c8000 (Abbott Diagnostics, Illinois, USA).

### Statistical analysis

The normal distribution of continuous variables was assessed using the Shapiro-Wilk test. Data were presented as mean ± standard deviation for normally distributed continuous variables and median (interquartile range) for non-parametric continuous variables, while categorical data were presented as percentages. Parametric (t-test) and non-parametric (Mann-Whitney) statistical tests were used. A reference interval was calculated using the Clinical and Laboratory Standards Institute guidelines for estimating percentiles and their 95% confidence intervals. Spearman coefficients were used to evaluate correlations between PLR and other variables (14). Statistical analysis was performed using MedCalc 12.1.4.0 statistical software (MedCalc® Statistical Software version 19.5.1 (MedCalc Software Ltd, Ostend, Belgium; https://www.medcalc.org; 2020)). Differences were considered significant if P (2-tailed) <0.05.

### Ethics

This study was approved by the Ethics Committee of the Institute of Public Health of Vojvodina, and the principles outlined in the Declaration of Helsinki were adhered to.

## Results

Table 1 presents the general characteristics of the examinees. The group consisted of 4672 residents of the Autonomous Province of Vojvodina. Analysing the gender distribution, male examinees outnumbered females: 3,909 (83.7%) of the examinees were male, while 763 (16.3%) were female. The average age was 43±10, ranging from 18 to 90 years.

**Table 1:** General characteristics of the study group. Continuous variables are expressed as median (interquartile range) and categorical data as percentages (%). WC - waist circumference; BMI - body mass index; SBP - systolic blood pressure; DBP - diastolic blood pressure; RBC - red blood cell; HCT - hematocrit; Hgb - hemoglobin; WBC - white blood cell; PLR - platelet-to-lymphocyte ratio

Parameter<br>N=4672	
Age (years)<br>Young age (18-39)<br>Middle age (39-64)<br>Elderly (65-90)	<br>1910/4672, 40.9%<br>2523/4672, 54%<br>239/4672, 5.1%
Sex (n/N, %)<br>Male<br>Female	<br>3909/4672, 83.7%/<br>763/4672, 16.3%
WC (cm)	92 (83-101)
Abdominal obesity (n/N%), Male	1115/3910, 28.5%
Abdominal obesity (n/N, %), Female	198/763, 25.9%
BMI (kg/m^2^)	26.6 (24.2-29.7)
Obesity, (n/N, %)	1096/4672, 23.5%
SBP (mmHg)	132.0 (120.0-146.0)
DBP (mmHg)	86.0 (79.0-94.0)
Hypertension (n/N, %)	788/4672, 16.9%
Glucose (mmol/L)	5.5 (5.1-6.0)
RBC (x10^6^/L)	5.05 (5.1-6.0)
HCT (g/L)	0.45 (0.42-0.47)
Hgb (L/L)	151.0 (143.0-158.0)
WBC (x10^9^/L)	6.8 (5.8-7.9)
Granulocyte (x10^9^/L)	4.1 (3.4-5.0)
Lymphocyte (x10^9^/L)	2.3 (1.9-2.7)
PLT (x10^9^/L)	249.0 (218.0-283.0)
PLR	109.0 (90.0-131.0)

**Figure 1:**
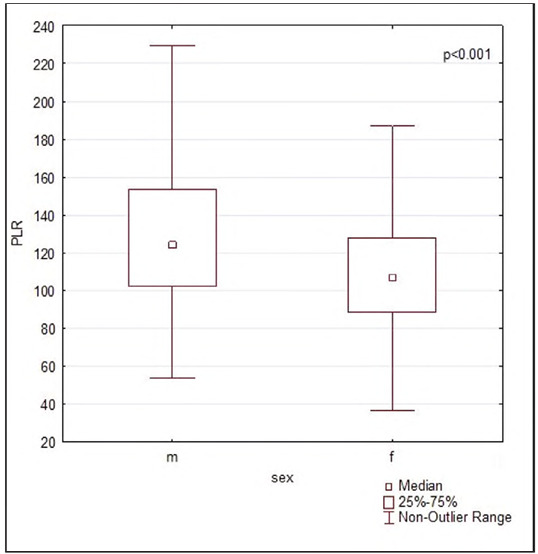
Platelet-to-lymphocyte ratios according to gender.<br>*Legend*: PLR - platelet to lymphocyte ratio; w - women; m - man<br>P<0.001, a statistically significant difference (Mann-Whitney)

Median PLR value was 109 [90.0-131.0]. Women had significantly higher levels of PLR compared to men [123 (101-152) vs. 105 (87-127), P<0.0001], as shown in Figure 1.

In women, the reference interval ranged from 70 (90%, CI: 65-74) to 231 (CI: 213-247), while in men, it was found to be lower, ranging from 61 (90%, CI: 60-63) to 183 (CI: 178-187).

Compared to women, men had: significantly higher BMI values [26.8 (24.7-30.1) vs. 23.9 (21.527.5), P<0.0001], significantly higher waist circumference [95 (86-104) vs. 78 (71-88), P<0.0001] as well as significantly higher SBP [134 (123-148) vs. 121 (110-134), P<0.0001] - Table 2. Statistically significant negative correlation between PLR and BMI was determined (r=-0.1, P<0.0001), WC (r=-0.1, P<0.0001) and SBP (r=-0.07, P<0.0001).

**Table 2:** The difference of the examined parameters in relation to gender.

Parameter N= 4672	Male (3909)	Female (763)	P
WC (cm)	95 (86-104)	78 (71-88)	<0.0001
BMI (kg/m^2^)	26.8 (24.7-30.1)	23.9 (21.5-27.5)	<0.0001
SBP (mmHg)	134 (123-148)	121 (110-134)	<0.0001

**Figure 2:**
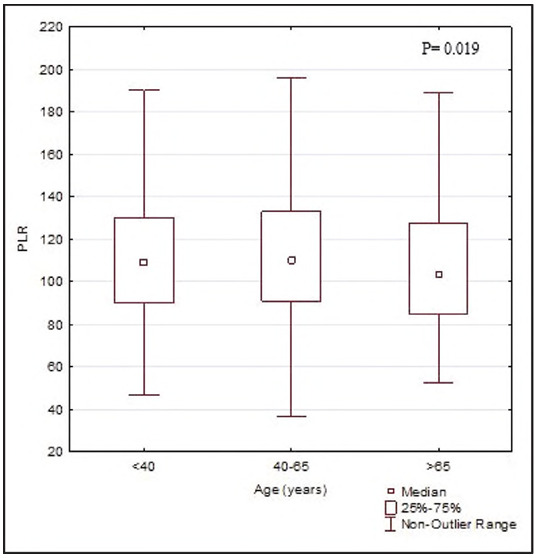
Platelet-to-lymphocyte ratios in age category.<br>*Legend*: PLR - platelet to lymphocyte ratio<br>P=0.019, a statistically significant difference (Kruskal-Wallis test)

For the purpose of analysing the influence of age to the values of PLR index, examinees were divided into three groups: first group (young age) consisted of those aged from 18 to 39, second (middle age) consisted of those aged from 40 to 46, while third group, the oldest one, (elderly) consisted of examinees older than 65. Statistical analysis showed that the PLR values are significantly lower in the elderly compared to examinees of middle and young age [103 (85.2-124) vs. 110 (91-133), vs. 190 (90130), P=0.019] (Figure 2).

In the second group, the average age was 46±14 years, ranging from 25 to 70 years old. The gender distribution was 52 men and 43 women. PLR index values, shown in Table 3, were not significantly different compared to the first group [108.0 (87136) vs. 109.0 (90.0-131.0)]. The median value of CRP for the second group was found to be 0.8 (0.21.5) ng/mL.

**Table 3:** Platelet to lymphocyte ratio obtained in two groups of subjects (n=4672 and n=95), expressed in mean, SD, median and lower/upper limits of the median (and their 95% CI).

	Initial cohort	Control cohort
Mean	114.8	114.7
SD	33.6	38.5
Median	109	108*
Lower limits of the median	62	62.5
95% CI	61-63	50-76
Upper limits of the median	194	182
95% CI	188-198	168-255
25th percentile	90	87
75th percentile	131	136
Min	36	46
Max	294	291

## Discussion

This is the first study in the northern region of the Republic of Serbia to determine the range of normal PLR values in a large cohort of clinically healthy individuals. Based on our results, which included 4,672 examinees, the 95% reference range for the PLR index was 70-231 for women and 61-183 for men. The established reference range was further validated in a second group of examinees (n=95), in whom the potential presence of an acute inflammatory process was excluded, as indicated by very low CRP levels (median 0.8 mg/L, range 0.2-1.5 mg/L).

A study conducted in North China reported lower 95% reference ranges for PLR (men: 36.63-149.13; women: 43.36-172.68) compared to those determined in our study [15]. Similarly, Lee et al. reported an average PLR value of 132.4, which is higher than the mean value observed in our cohort (114.8) [16]. Kweon et al. also reported an average PLR value of 121.07 (17). Our study identified gender-based differences in PLR index values, with females exhibiting significantly higher PLR values than males. Similar findings were reported in 2019 (108.02±32.99 for females vs. 92.88±28.70 for males) [17]. Since the PLR index is calculated by dividing the platelet count by the lymphocyte count, higher values in females may be attributed to their generally higher platelet counts compared to males [18] [19] [20]. Lower serum iron concentrations in females, particularly during the reproductive period, may stimulate platelet production, resulting in higher absolute platelet counts [21] [22].

Additionally, our study found that the lowest PLR values occurred in examinees older than 65 years. This may be explained by reduced reserves of hematopoietic stem cells, resulting in decreased thrombopoiesis. These findings are consistent with previously published data [23].

Obesity may represent a significant confounding factor in clinical studies examining platelet and/or lymphocyte counts, given the potential presence of low-grade inflammation in the pathophysiology of the disease itself. In studies conducted by Shuang Han and Erdal, obesity was associated with increased platelet parameters and a higher PLR index [24] [25]. Their findings reported a notable incidence of obesity (23.5%) and abdominal obesity within the study population. Among males, the incidence of abdominal obesity was 28.5%, compared to 25.9% in females.

In contrast, our results demonstrated a low-level and inverse correlation between PLR and obesity indicators. Differences in the metabolic phenotype of obese individuals may explain the discrepancy between our findings and those previously published. Specifically, an unhealthy metabolic phenotype - characterised by insulin resistance, hyperglycemia, hyperlipidemia, and arterial hypertension - may have a direct and significant impact on PLR values. In our study population, no glucose metabolism disorders were confirmed, and the incidence of arterial hypertension was relatively low (16.9%).To determine the cut-off values at which inflammation can be predicted, it would be necessary to assess normal or reference values of the PLR index. Analysed published data suggest that different reference values should be set according to the race, age and gender. Hence, the results of this study could be fundamental in predicting inflammatory processes within our population. The basic limitation of this study is the relatively low number of female and elderly examinees.

In a representative sample of examinees, primarily consisting of young and middle-aged adults from the northern region of the Republic of Serbia, the reference interval for the PLR index was determined to be 70-231 in females and 61-183 in males. Individuals older than 65 years may have lower PLR values. Further research is needed to confirm the clinical significance of these reference values.

## Dodatak

### Acknowledgements

This study was supported by the Provincial Secretariat for Science and Technological Development, Autonomous Province of Vojvodina, Republic of Serbia, under grant No. 114- 451-2013/2016-01.

### Conflict of interest statement

All the authors declare that they have no conflict of interest in this work.

### List of abbreviations

CBC, Complete blood count;<br>NLR, neutrophils to lymphocytes ratio;<br>PLR, platelets to lymphocyte ratio;<br>PLT, platelet
